# DNA Methyltransferases Contribute to Cold Tolerance in Ticks *Dermacentor silvarum* and *Haemaphysalis longicornis* (Acari: Ixodidae)

**DOI:** 10.3389/fvets.2021.726731

**Published:** 2021-08-26

**Authors:** Desmond Onyeka Agwunobi, Miao Zhang, Xinyue Shi, Shiqi Zhang, Meng Zhang, Tongxuan Wang, Abolfazl Masoudi, Zhijun Yu, Jingze Liu

**Affiliations:** Hebei Key Laboratory of Animal Physiology, Biochemistry and Molecular Biology, College of Life Sciences, Hebei Normal University, Shijiazhuang, China

**Keywords:** DNA methyltransferase, epigenetics, *Dermacentor silvarum*, *Haemaphysalis longicornis*, cold tolerance

## Abstract

DNA methylation, mediated by DNA methyltransferases (Dnmts), is a typical epigenetic process that plays an important role in affecting organism acclimatization and adaptation to environmental changes. However, information about Dnmts and their associations with the cold tolerance of ticks remains meager. Hence, in the present study, the Dnmts in important vector ticks *Dermacentor silvarum* and *Haemaphysalis longicornis* were cloned and identified, and their functions in cold response were further explored. Results showed that the length of *DsDnmt* and *DsDnmt1* in *D. silvarum*, and *HlDnmt1* and *HlDnmt* in *H. longicornis* were 1,284, 549, 1,500, and 1,613 bp, respectively. Bioinformatics in protein analysis revealed that they were all unstable hydrophilic proteins and were mainly characterized with Dcm (DNA cytosine methyltransferase domain), Dnmt1-RFD (DNA methyltransferase replication foci domain), zf-CXXC (zinc finger-CXXC domain), and BAH (Bromo adjacent homology domain). The relative expression of these *Dnmts* was reduced after cold treatment for 3 days (*P* < 0.05), and increased with the extension of treatment. Western blot revealed that Dnmt1 decreased first and then increased significantly (*P* < 0.05) in both tick species, whereas other Dnmts fluctuated at varying degrees. RNA interference significantly silenced the genes *Dnmts* (*P* < 0.01), and mortality increased significantly (*P* < 0.05), when exposed to sub-lethal temperature, underscoring the important roles of Dnmts during the cold response of *D. silvarum* and *H. longicornis*. The above results lay the foundation for further understanding of the epigenetic regulation of DNA methylation in cold acclimatization and adaptation of ticks.

## Introduction

Ticks are obligate blood-sucking ectoparasitic arthropods, which can spread a variety of pathogens and cause zoonotic diseases (1). They are considered as the most important vector of pathogens of domestic and wild animals, and only second to mosquitoes as vectors of human disease (2). With social and economic development and changes in the ecological environment, humans have more contact with ticks which has led to an increase in the incidence of tick-borne diseases (1). Ticks and tick-borne diseases have raised public health issues and posed a serious threat to animal husbandry development. Recently, ticks have been implicated as the vector of Songling virus (SGLV) (3) and Alongshan virus (ALSV), both associated with human febrile illness (4, 5), and severe fever with thrombocytopenia syndrome virus (SFTSV), the causative agent of severe fever with thrombocytopenia syndrome (SFTS) (6).

The tick *Dermacentor silvarum* Olenev is widely distributed in Russia, Mongolia, and the north of China (7, 8), and has been implicated as the vector and reservoir for the maintenance of tick-borne encephalitis virus through transstadial and transovarial transmission in China (9). *Rickettsia raoultii, R. slovaca*, and *R. heilongjiangensis* (spotted fever group rickettsiae), along with *Ehrlichia chaffeensis* (the causative agent of human monocyclic *Ehrlichia*), have been identified in *D. silvarum* (10, 11). *D. silvarum* is also the primary vector of two babesiosis-causing parasites, *Babesia equi* and *B. caballi*; and the North Asian tick typhus pathogen known as *R. sibirica* (1). The tick *Haemaphysalis longicornis* Neuman, is one of the most predominant tick species in Asia countries and a serious pest of livestock in the Australasian and Western Pacific Regions (12, 13). *H. longicornis* has expanded rapidly to many states in the USA since its first identification in New Jersey (14–16). It has been implicated as a vector of several pathogenic agents including the causative agent (*Theileria sergenti* and *T. buffeli*) of hemolytic anemia in wild and domestic cattle (17), causative agent (*T. orientalis*) of bovine theileriosis in Australia, and New Zealand (18), and causative agent (*Babesia* spp.) of livestock babesiosis (19). Moreover, it can transmit many spotted fever group rickettsiae (SFGR) such as *R. japonica, R. heilongjiangensis, R. raoultii*, and *Candidatus Rickettsia tarasevichiae* (12, 20–22). More recently, the severe fever with thrombocytopenia syndrome virus (SFTSV) was also identified in *H. longicornis* from South Korea, Japan, and China, which has caused great damages to livestock production and human health (6, 23, 24).

Many terrestrial arthropods are frequently faced with temperatures low and sustained enough to freeze their body fluids and therefore, have implemented some strategies to mitigate this risk (25, 26). Some of these strategies include the modification of body fluid constituents to prevent ice crystallization (27), cryoprotective dehydration (28), freeze avoidance, behavioral avoidance, and freeze tolerance which involves the conversion of about 82% of body water into internal ice (26, 29). Although freeze-tolerant arthropods utilize many potentially protective molecules, there is no obvious molecule that appears to be sufficient to support this cold-tolerance strategy (26). Climate variables are important factors affecting tick distribution (30), which also influence tick survival, development, and activity during off-host periods (31, 32). Hence, the ambient temperature, especially the low temperatures in winter, could play a significant role in determining the host-seeking behavior of ticks. A complex physiological and biochemical process is involved in the response of ticks to low temperatures, including changes in the intracellular environment and substance synthesis, as well as the expression and regulation of some temperature-stress-related genes (33, 34).

DNA methylation is an epigenetic modification that is heritable and reversible, which could play some roles in the mediation of rapid plastic responses of organisms to environmental stress (35, 36). The methylation of DNA at the fifth position of cytosine (5 mC) and the sixth position of adenine (6 mA) influences gene expression by the regulation of the chromatin architecture and gene transcription (37, 38). The capacity of organisms to adapt to environmental perturbations is increased by DNA methylation (39). This mechanism is mediated by a vital group of evolutionarily conserved enzymes known as DNA methyltransferases (Dnmts), which catalyze the methylation of DNA at cytosine residue to form 5-methylcytosine (40). Though mounting evidence has highlighted that variation in DNA methylation patterns could contribute to the acclimation and adaptation of organisms to different environmental stresses (41–43), the relationship between Dnmts and the cold tolerance of ticks remains poorly understood.

In the present study, the DNA methyltransferases in *D. silvarum* and *H. longicornis* were cloned and identified, and their functions in the cold tolerance of ticks were further explored via RNA interference. Western-blot methods were used to investigate the expression dynamics of DNA methyltransferases of two ticks under cold stress. The findings of this study provide insight into the regulatory role of DNA methylation in the cold response of these two tick species, and lay the foundation for further investigation on the epigenetic and molecular regulatory mechanisms underlying the cold tolerance of ticks.

## Materials and Methods

### Ticks

The unfed adult ticks of *Dermacentor silvarum* and *Haemaphysalis longicornis* were collected by flag-dragging from the vegetation of Xiaowutai National Nature Reserve, Zhangjiakou City, Hebei Province (114°47'−115°28'E, 39°50'– 40°6'N). They were brought back to the laboratory and put on the ears of the New Zealand white rabbits for feeding, and cultured in an environmental chamber (26 ± 1°C, relative humidity 75 ± 5%, 16 h of light: 8 h in darkness) during the non-parasitic period (44). The second-generation adult ticks that molted for two weeks were used in subsequent tests. All experiments were approved by the Animal Ethics Committee of Hebei Normal University (Protocol Number: IACUC-176810).

### RNA Extraction and cDNA Synthesis

Total RNA was extracted from ticks (each group contains 10 *D. silvarum* or 20 *H. longicornis*) using TRIzol reagent (Invitrogen, Carlsbad, CA, USA) following the manufacturer's protocol. RNA quantification was carried out using Nano Drop® ND-1000 Spectrophotometer (Thermo Fisher Scientific, Waltham, MA, USA), with a ratio of A260/A280 typically above 2.0, and the RNA quality was evaluated by 1% agarose gel electrophoresis. Then, 2.0 μg of each extracted RNA was used in a 20.0 μL reaction volume with an oligo (dT)_18_ primer, and reverse transcription was performed to synthesize cDNA following the instructions provided with the TransScript® One-Step gDNA Removal and cDNA Synthesis SuperMix (TransGen Biotech Co., Ltd, Beijing, China). Primers (Table 1) were designed using DNAMAN (Lynnon Biosoft, San Ramon, CA, USA) and Primer Premier version 5.0 for Windows (Premier Biosoft International, Palo Alto, CA, USA), based on an alignment of several sequences obtained from the National Center of Bioinformatics Information (NCBI) website (http://www.ncbi.nlm.nih.Gov) and transcriptomic assembled sequences (Bio-Project PRJNA565692). The PCR conditions include an initial 2 min denaturation at 94°C, 35 cycles of 30 s at 94°C, 30 s at the melting temperature *T*_m_ (58–60°C) of the gene-specific primers, and 30 s at 72°C, and then a final extension at 72°C for 10 min carried out on an Applied Biosystems® Veriti® 96-Well Thermal Cycler (Life Technologies Ltd, Marsiling, Singapore). The amplified fragments were verified and separated on a 1% agarose gel. Bands with expected sizes were excised and purified by EG101-01 EasyPure Quick Gel Extraction Kit (TransGen, Beijing, China) according to the manufacturer's protocol. The purified products were sequenced and used for subsequent analysis.

**Table 1:** Primer sequences used for cDNA cloning, real-time quantitative PCR, and dsRNA synthesis.

**Tick species**	**Gene name**	**Primer sequence (5^**′**^-3^**′**^)**
**Primers of DNA methyltransferase genes for PCR**		
*Dermacentor silvarum*	*Dnmt*	F_1_:CTCGCAGAGTGCTCAACAGG
		R_1_:GGCTTGTTCTTCAGCGTGG
		F_2_:GTGAACACCACGGCGAACT
		R_2_:TCACTGAGGCTGAGGAAACTTG
		F_3_:TGGCTACCACATTCATCGTTA
		R_3_:GCAGCATTTCCAGGACATCT
		F_4_:TCAAGTTTCCTCAGCCTCAGTG
		R_4_:GATGAAATAAGGTCCCCGAGAG
	*Dnmt1*	F:GGTCGGACGAGAGGTTTTTC
		R:GGGAAACCTGGGCAAAACT
*Haemaphysalis longicornis*	*Dnmt1*	F_1_:GTCGTTTCCGCACTTCGTAG
		R_1_:GTTGAACTTGGCAAAAGGGTAG
		F_2_:TCAAGCGACCTGAAGGAATG
		R_2_:TTCATCCTATGGCTCCGTAAAG
		F_3_:GGTTCCTGATTGTGCGAGA
		R_3_:TGTTCAATGGCCTTCGTCT
		F_4_:GCAGAAGAGCAGCCGCAT
		R_4_:CTCCTTGTTTCTGAACTGCTCG
	*Dnmt*	F_1_:AGTGTGTTCAACGACGGGG
		R_1_:TCGATCACCAGCTTGCTCAT
		F_2_:TATCGACGCCATCAAGGAGA
		R_2_:GCCGCCAAACTTCACCAT
		F_3_:AAGCGTTGCGGAGTCTGTG
		R_3_:GTGCCCCTCCAAAACCAGT
		F_4_:GACCCTGGAACCCCACTCTAT
		R_4_:CCGCTCTCTTCTTCGACAACT
**Primers for Real-time quantitative PCR**		
*Dermacentor silvarum*	*Dnmt*	F:TGTCAAGTTTCCTCAGCCTCA
		R:CGACATAATGACCATAGCCCTT
	*Dnmt1*	F:AAGAACTCGCTGGTGTCATCGT
		R:TCCAGAGTCAGCGGCAACAT
	*Actin*	F:TTCCAGCCCTCGTTCCTGGGTAT
		R:AATGATCTTGATCTTCATGGT
*Haemaphysalis longicornis*	*Dnmt1*	F:GTGGCTGATGAAGGCAAAGA
		R:CGGCAGAGTTCAAGCAGGT
	*Dnmt*	F:TCGTCAATGAATGCGAGAACC
		R:AGAACGACTTGCCGTCATCATC
	*Actin*	F:CGTTCCTGGGTATGGAATCG
		R:TCCACGTCGCACTTCATGAT
**dsRNA synthesis primers**		
*Dermacentor silvarum*	*Dnmt*	F:GCGTTCAACTTCCCCGACA
		R:GCCAGGCAGTAGAAACGCATT
	*Dnmt1*	F:GCGATGTGCTAAAGGGAAAGA
		R:CTGAAGCGAGGCAAAGGTG
	*Dnmt1*	F:CAACACCCCTGAAGTTAGCAA
		R:TCAGCCACTCTTCGCCAA
	*Dnmt*	F:TCGCCAAGTTCACGGAGGA
		R:TGCCGCCAAACTTCACCAT

*Oligonucleotide primers for the synthesis of dsRNA comprised of T7 promoter sequences at the 5′ end*.

### Bioinformatics Analysis of DNA Methyltransferase

Sequence alignment and identity analyses were carried out using DNAMAN (Lynnon Biosoft, San Ramon, CA, USA) and BLASTn (http://www.ncbi.nlm.nih.gov/BLAST). The identification of Open reading frames (ORFs) was carried out using ORF Finder (http://www.ncbi.nlm.nih.gov/gorf/orfg.cgi), and a conserved domain search using the Conserved Domain Database (CDD) v.3.16 of NCBI was performed (45). The complete genome sequence of the DNA methyltransferases of *D. silvarum* and *H. longicornis* were deposited in the NCBI nucleotide database under the accession numbers: **MW528395** (*DsDnmt*), **MW528396** (*DsDnmt1*), **MW528397** (*HlDnmt1*), and **MW528398** (*HlDnmt*). The physicochemical properties of DNA methyltransferase proteins including the molecular weights and theoretical isoelectric points (pI) were calculated using the Compute pI/Mw tool (http://web.expasy.org /protparam/) and DiANNA website (http://bioinformatics.bc.edu/clotelab/DiANNA/). The hydrophobic properties (hydrophobicity) of the putative DNA methyltransferases were determined using Hphob/Kyte&Doolittle model (46). The prediction of the protein tertiary structures was carried out with the Swiss-Model web server (http://swissmodel.expasy.org/).

To reconstruct the evolutionary relationships of the DNA methyltransferase genes, phylogenetic analysis was performed using Molecular Evolutionary Genetics Analysis (MEGA) software version 6.06 (47) based on the amino acid sequences retrieved from the NCBI protein database. Alignment of sequences was carried out with the ClustalW method, and the phylogenetic tree was obtained by the Neighbor-Joining method (48) with complete gap deletion and a bootstrap value of 100% in 1,000 replicates. Distances were computed using the Poisson correction method (49) and were in units of the number of amino acid substitutions per site.

### Synthesis of dsRNA of Target Genes

The primers (Table 1) for RNA interference of DNA methyltransferase genes were designed using DNAMAN (Lynnon Biosoft, San Ramon, CA, USA) and Primer Premier Version 5.0 for Windows (Premier Biosoft International, Palo Alto, CA, USA). Oligonucleotide primers comprising T7 promoter sequences at the 5′ end for *in vitro* transcription and synthesis of double-strand RNA (dsRNA) were used to amplify cDNA encoding the DNA methyltransferases. All the oligonucleotide primers used for this study were synthesized by Invitrogen^TM^ (Beijing, China). *In vitro* dsRNA synthesis was carried out using the T7 RiboMAX^TM^ Express RNAi System (Promega, Madison, USA). The transcription reaction contained 1 μL of cDNA as the transcription template, 7.0 μL of RNase-free water, and 2 μL of T7 enzyme mix in 10 μL Express 2x Buffer culminating to a final volume of 20.0 μL.

The reactions were carried out at 37°C for 30 min, 70°C for 10 min, and allowed to stand for 20 min at room temperature. After adding 2 μl of RQ1 RNase-Free DNase and 2 μL of diluted RNase A solution, the reaction was incubated at 37°C for 30 min. Then, 4.4 μL Sodium Acetate and 110 μL 95% ethanol was added and incubated on ice for 5 min, followed by centrifugation at 16,000 g for 5 min at 4°C. The precipitate was added 500 μL of 70% ethanol (pre-cooled at −20°C), resuspended and centrifuged at 10,000 g for 5 min at 4°C, and the precipitate was dried at room temperature for 15 min. Finally, the dsRNA pellet was resuspended in diethylpyrocarbonate (DEPC)-treated water. The concentration and quality of dsRNA were determined by Nano Drop® ND-1000 Spectrophotometer and 1% agarose gel electrophoresis. The dsRNA was then stored at −80°C until use.

### RNA Interference

The procedure for the injection of dsRNA into 40 female ticks (20 *D. silvarum* and 20 *H. longicornis*) was performed according to (50). After surface-sterilization of the ticks, 2 μL of dsRNA solutions were injected into the hemocoel of unfed female ticks through the third and fourth coxa at a concentration of 1 μg/tick using a 10 μL Microliter^TM^ Syringes (Hamilton, Nevada, USA). The control groups were injected with dsRNA-GFP. After injection, the ticks were placed in the environmental incubator (temperature 26 ± 1°C, relative humidity 75 ± 5%, 16 h of light: 8 h in darkness) for 24 h. Each treatment was carried out in three replicates.

The total RNA was extracted from the ticks using TRIzol reagent, and their cDNA was synthesized as described previously. The mRNA expression of the target genes after knockdown with dsRNA was evaluated by quantitative real-time PCR (qRT-PCR), performed using an Mx3005P qPCR system (Agilent Technologies, Santa Clara, USA) using TransStart® Top Green qPCR SuperMix (TransGen Biotech) according to the manufacturer's instructions. The real-time PCR assays were prepared using a 96 well plate (Axygen, California, USA) with 10 μL of 2 × *TransStart*® Top Green qPCR SuperMix, 1.0 μL of the cDNA template, 0.4 μL of each forward and reverse gene-specific primers (Table 1), and 0.4 μL of Passive Reference DyeII in a final volume of 20.0 μL. The conditions of the thermocycler used were set at 94°C for 30 s, followed by 40 cycles of 94°C for 5 s and 60°C for 30 s. Then, 95°C for 1 min, 55°C for 30 s, and a final 95°C for 30 s. Each sample was assessed in triplicate (technical replicates). Control without the cDNA was included in all batches. *Actin* was used as the reference gene given that it is a housekeeping gene that is constitutively expressed under various temperature stress conditions (40). The relative expressions of the DNA methyltransferase genes were calculated by a 2^−Δ*ΔCT*^ method (51).

### Cold Tolerance of Ticks After RNAi

After confirmation of the knockdown of DNA methyltransferase genes, *D. silvarum* and *H. longicornis* ticks were exposed to a sub-lethal temperature of −22 and −14°C, respectively, for 2 h in a low-temperature thermostatic bath (Meixiang Instrument Co. Ltd., Shanghai, China). The temperature of −22°C was selected given that the discriminating temperatures (resulting in 20% survival) for the females and males were −21.7 and −22.6°C, respectively (52). Then the ticks were removed from the thermostatic bath, and the mortality rate was calculated. Ticks were considered dead if they cannot coordinate their appendage after stimulated by CO_2_.

### Relative Expression of DNA Methyltransferase Proteins Under Different Cold Treatments

The unfed adult *D. silvarum* or *H. longicornis* were divided into 6 groups with each group comprising 20 ticks. Three groups kept at the normal environment chamber (26°C) served as control; whereas the other three groups (experimental group) were exposed to low temperature (4°C) for 3, 6, and 9 days, respectively. After treatment, the ticks were grounded to powder in liquid nitrogen and transferred to a pre-cooled 1.5 mL Eppendorf (EP) tube containing 1 mL of sterile phosphate-buffered saline (PBS, pH 7.4) and 10 μL of phenylmethylsulfonyl fluoride (PMSF) protein inhibitor. Then, it was vortexed and centrifuged at 13,000, rpm for 10 min at 4°C. The supernatant was transferred to a pre-cooled 1.5 mL EP tube. Protein concentration was measured using BCA (Bicinchoninic Acid) Protein Assay Kit (CWBIO, Haimen, Jiangsu, China), following the manufacturer's recommendations.

The extracted protein samples were dissolved in 0.1 M Tris-HCl (pH 6.8) containing 2% SDS, 5% 2-mercaptoethanol, 10% glycerol, and 0.05 % bromophenol blue, followed by 2 min boiling. The SDS-PAGE was performed on 12% separating gels with 4% stacking gels containing 0.1% SDS using PAGE Gel Preparation Kit (Epizyme, USA). A 0.05 M Tris-glycine buffer (pH 8.3) containing 0.1% SDS was used to perform electrophoresis at a constant voltage of 120 V until the tracking dye reached the end of the gel. Subsequently, the electro-transfer onto polyvinylidene difluoride (PVDF) membrane was carried out at 22V for 25 min using a Trans-Blot SD apparatus (BIO-RAD, Hercules, CA, USA). The membrane was blocked for 2.5 h with 5% skim milk in 1xTBST (Tris-buffered saline with 0.1% Tween® 20 detergent) solution, washed three times with 1xTBST in 15 min, and then incubated overnight with a monoclonal anti-DNMT antibody (1: 2000) (GeneTex, TX, USA). After three washes with 1xTBST in 15 min, the membrane was incubated with horseradish peroxidase (HRP)-conjugated goat anti-mouse IgG for 2 h at room temperature, the signal was detected by ultra-sensitive enhanced chemiluminescent (ECL) substrate using SuperSignal^TM^ West Femto Trial Kit (Thermo Scientific, Waltham, MA, USA), visualized and analyzed with an Image Lab Software-controlled Gel Doc^TM^ XR+ System (BIO-RAD).

### Statistical Analysis

The differences among different treatment groups were compared with one way ANOVA test, and Tukey's test was used for *post hoc* analysis. A significant difference is defined as *P* < 0.05. Data analysis was carried out using Graph Prism version 8.0.2 for Windows (GraphPad Software, Inc., San Diego, USA).

## Results

### Cloning of DNA Methyltransferase Genes

The cDNA obtained from the total RNA of *D. silvarum* and *H. longicornis* were used as a template with specific primers for PCR amplification of the target DNA methyltransferase gene. The amplified products subjected to agarose gel electrophoresis showed single, clear, and bright bands (Supplementary Figure 1). The target fragments of *D. silvarum* DNA methyltransferase gene 1 were 493, 456, 552, and 539 bp, while the target fragment for DNA methyltransferase gene 2 was 549 bp. The target fragments of *H. longicornis* DNA methyltransferase gene 1 were 433, 851, and 526 bp, whereas the target fragments for gene 2 were 389, 517, 470, and 467 bp. After the blast, the two DNA methyltransferases of *D. silvarum* showed the highest sequence identity with the DNA cytosine methyltransferase (*Dnmt*) of *Ixodes ricinus* (65%) and DNA (cytosine-5)-methyltransferase (*Dnmt1*) of *Ixodes scapularis* (64%), whereas the two DNA methyltransferases of *H. longicornis* showed the highest sequence identity with the DNA methyltransferase 1 (*Dnmt1*) (81%) and DNA (cytosine-5)-methyltransferase PliMCI (*Dnmt*) (77%) of *Ixodes scapularis*. Thus, the DNA methyltransferase of *D. silvarum* and *H. longicornis* were named *DsDnmt* (1,284 bp), *DsDnmt1* (549 bp), *HlDnmt1* (1,500 bp), and *HlDnmt* (1,613 bp), respectively. The multiple sequence alignments indicate that the deduced amino acid sequences of DsDnmt, DsDnmt1, HlDnmt1, and HlDnmt showed high sequence identity to their corresponding sequences (Supplementary Figure 2).

### Sequence Analysis

The *DsDnmt* had a relative molecular mass of 391957.00 Daltons with a base ratio of A+T at 5.56% and a base ratio of G+C at 54.44%. The predicted full length of its open reading frame (ORF) was 1,128 nucleotide (nt) ranging from 67 to 1,194 base pairs. *DsDnmt1* had a relative molecular mass of 166573.00 Daltons with the base ratio of A+T <44.99% and G+C more than 55.01%. Its coding sequence (CDS) had a full length of 549 nt (1 to 549 bp). The sequence lengths of *HlDnmt1* and *HlDnmt* were 1,500 and 1,613 bp, respectively, with multiple replications start sites. The total ORF length of *HlDnmt1* was 1,464 nt (17 to 1,480 bp) with a relative molecular mass of 458465.00 Daltons and a base ratio of 37.87 and 62.13% for A+T and G+C, respectively. The relative molecular mass of *HlDnmt* mRNA was 491394.00 Daltons with a base ratio of 38.19% for A+T and 61.81% for G+C. The total length of the CDS was 1,611 nt and it ranges from 1 to 1,611 bp.

### Protein Composition Analysis

#### Analysis of Amino Acid Composition

The predicted protein sequence of *DsDnmt* constitutes 375 amino acids, of which there were 19 types of amino acids. Leu accounted for 12.27%, followed by Ser, Pro, and Arg which accounted for 8.27, 7.47, 7.20, and 7.47%, respectively, whereas the least content was methionine which accounted for 1.87%. The total number of positively charged residues (Arg+Lys) was 38 and negatively charged residues (Asp+Glu) was 42. The predicted protein sequence of *DsDnmt1* fragment contains 183 amino acids, of which can be categorized into 20 types. The protein encoded by the *DsDnmt1* fragment contains 183 amino acids, of which there are 20 types of amino acids, most of which was Leu accounting for 13.11%. Gly accounted for 9.29%, while each of Ser and Arg, accounted for 8.20% and with the least content being Trp which accounted for 0.55%. The total number of positively and negatively charged residues are 22 and 12, respectively. The predicted protein sequence of *HlDnmt1* contains 487 amino acids, of which there are 20 types of amino acids. The most abundant amino acid was Ala accounting for 10.25%, followed by Leu, Arg, Glu, and Lys, accounting for 9.84, 9.63, 8.20, and 7.99% respectively, whereas the least abundant was Cys accounting for 0.82%. The residues that were positively charged were 86 amino acids while the negatively charged residues were 66 amino acids. There were 537 amino acids in the predicted protein sequence of *HlDnmt*, of which can be categorized into 20 types, with Val being the most abundant accounting for 8.75%. Asp, Glu, and Ala accounted for 8.57, 8.19, and 8.01%, respectively, whereas the least abundant, is Trp, which accounts for 1.30%. A total of 62 amino acids were positively charged while a total of 90 were negatively charged.

#### Prediction of Amino Acid Hydrophobicity

The hydrophobic properties of the DNA methyltransferases were determined with the Hphob/Kyte&Doolittle model by a window size of 9 amino acids. The maximum hydrophobic value for DsDnmt was 2.078, located at the 50th and 121st amino acids which were Ala and Phe, respectively, whereas the minimum was −2.911 at the 111th amino acid (Gln). For DsDnmt1, the maximum hydrophobic value was 2.233, which was the 172nd amino acid (Ala), while the minimum was −2.489, the 92nd amino acid (His). The maximum hydrophobic value for HlDnmt1 was 1.722 at the 412th amino acid (Ala) and the minimum value was −3.844 at the 223rd amino acid (Arg). HlDnmt had 2.044 as its maximum hydrophobic value at the 445th amino acid (Leu) and −2.900 as its minimum hydrophobic value at the 338th amino acid (Arg). The total average hydrophobicity of DsDnmt, DsDnmt1, HlDnmt1, and HlDnmt proteins are −0.348, −0.239, −0.728, and −0.459, respectively, suggesting that the four DNA methyltransferases were hydrophilic proteins (Supplementary Figure 3).

#### Prediction of the Protein Physical and Chemical Properties

The physicochemical properties of DNA methyltransferase protein were predicted through the ProtParam Expasy website (http://web.expasy.org/protparam/) and DiANNA website (http://bioinformatics.bc.edu/clotelab/DiANNA/). All four putative proteins were predicted to have a nuclear localization (Table 2). The instability index for all four studied proteins was higher than 40, indicating that they were unstable, whereas the predicted disulphide bonds of the proteins might help to maintain their molecular shape, stability, and substrate specificity (Table 2).

**Table 2:** Predicted properties of the DNA methyltransferase protein of *Dermacentor silvarum* and *Haemaphysalis longicornis*.

**Predicted properties**	**Protein name**			
	**DsDnmt**	**DsDnmt1**	**HlDnmt1**	**HlDnmt**
Total atomic number	5865	2846	7762	8241
Molecular weight	42061.62 Da	20329.35 Da	54862.47 Da	59598.01 Da
Theoretical isoelectric point (PI)	6.22	9.50	9.67	4.88
Hydrophobic Index	−0.348	−0.239	−0.728	−0.459
Instability index	55.11	50.26	55.33	53.27
Fat index	81.87	78.85	74.80	70.43
Location in the cell	Nucleus	Nucleus	Nucleus	Nucleus
Disulphide bond	10–41, 47–98	8–19, 76–81	161–197	36–272
	184–196	109–110	317–383	70–267
	204–208	141–149		194–515
	268–332			261–506
	269–336			264–470
				275–278
				293–446
				294–299
				434–502

#### Phylogenetic Relationship

**Figure 1:**
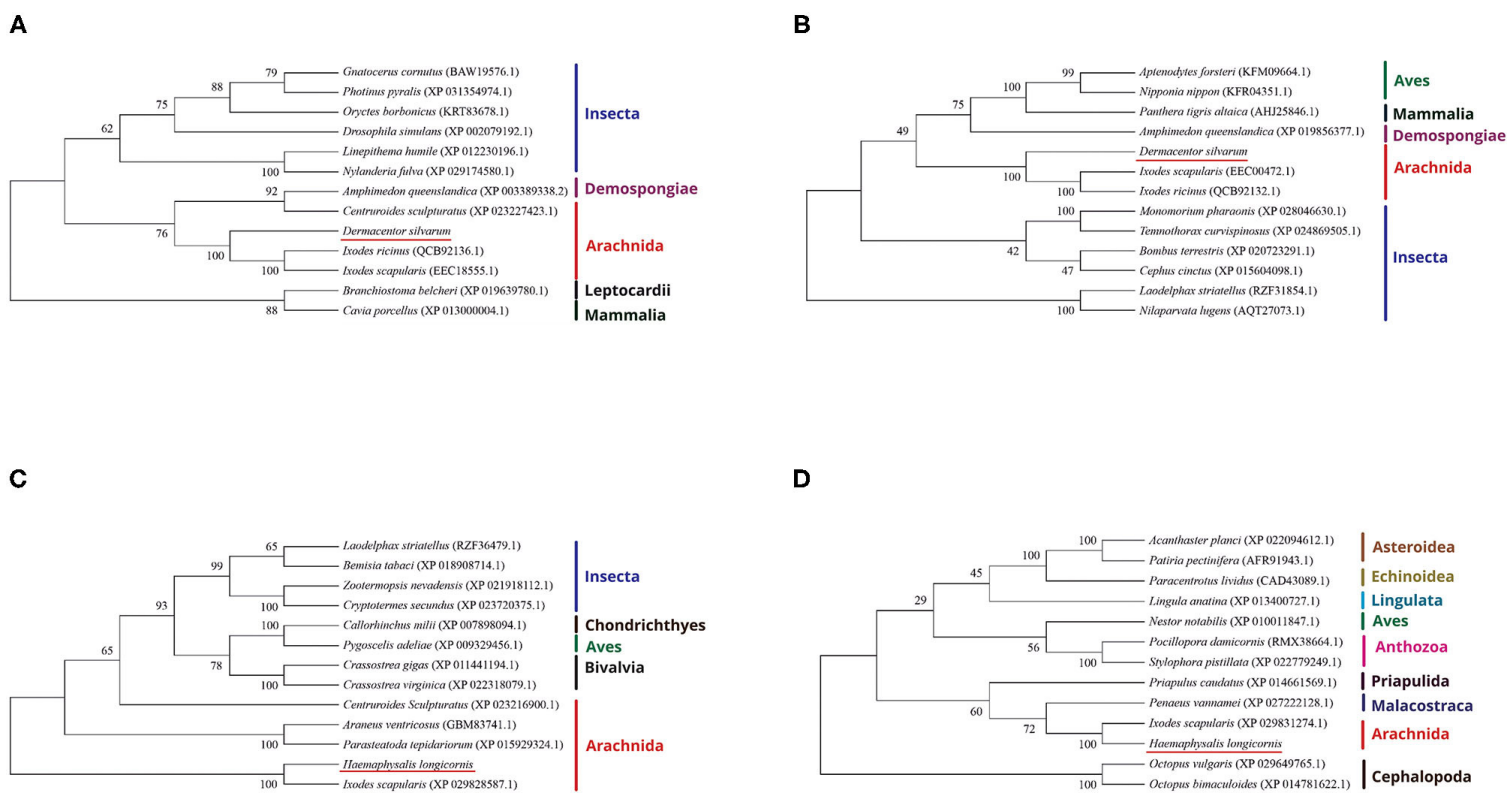
Phylogenetic reconstruction of DNA methyltransferases based on the neighbor-joining method. The listed species are colored according to their classification to particular taxonomic classes. **(A)** Illustration of the phylogenetic relationship of DsDnmt sequences to Dnmts of other species; **(B)** Illustration of the phylogenetic relationship of DsDnmt1 sequences to Dnmts of other species; **(C)** Illustration of the phylogenetic relationship of HlDnmt1 sequences to Dnmts of other species; **(D)** Illustration of the phylogenetic relationship of HlDnmt sequences to Dnmts of other species.

The evolutionary relationship between the DNA methyltransferases of *D. silvarum* and other species as well as those of DNA methyltransferases of *H. longicornis* were constructed (Figure 1). Results revealed that the DNA methyltransferases of *D. silvarum* and *H. longicornis* clustered the arthropod species into their corresponding taxonomic classes. The putative *DsDnmt* and *DsDnmt1* share the same node with the sequences of the closely related *I. ricinus* (QCB92136.1), *I. scapularis* (EEC18555.1), and *I. ricinus* (QCB92132.1), *I. scapularis* (EEC00472.1), respectively. Similarly, the putative *HlDnmt1* and *HlDnmt* share the same node with the sequence of the closely related *I. scapularis* (XP029828587.1, XP029831274.1). Ticks belong to a relatively independent branch (arachnids) and are shown to be closely related to other arachnids than the insects. Vertebrates such as mammals, birds, and fishes were placed in an independent branch indicating that their DNA methyltransferases are less closely related to those of ticks than mollusks and other marine organisms.

#### Conserved Domain Analysis

**Figure 2:**
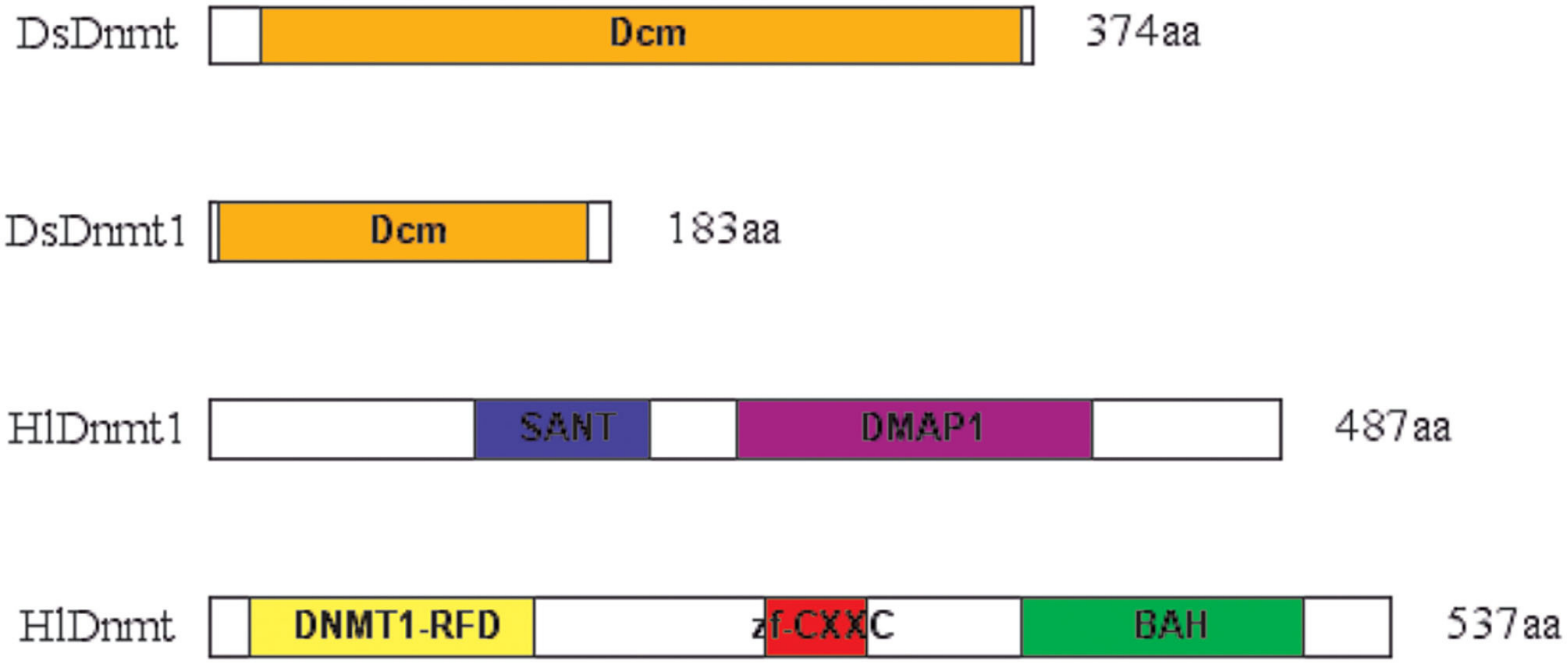
Schematic presentation of conserved domains identified for the putative DNA methyltransferases of *D. silvarum* and *H. longicornis*. Putative DsDnmt and DsDnmt1: orange—Dcm domain; putative HlDnmt1: blue—SANT domain, purple—DMAP1 domain; putative HlDnmt: yellow—DNA methyltransferase replication foci domain (DNMT1-RFD), red—CXXC zinc finger domain, green—Bromo adjacent homology (BAH) domain.

The conserved domain search was performed using the Conserved Domain Database (CDD) v.3.16 of NCBI with default parameters and a standard display mode. The result revealed that putative DsDnmt and DsDnmt1 contain DNA cytosine methylase (Dcm) domain (interval 24–369 bp and interval 5–172 bp, respectively) with multiple substrates- and DNA-binding sites (Figure 2). HlDnmt1 contains DMAP1 (DNA methyltransferase 1-associated protein) domain (interval 241–401 bp) and SANT domain (interval 121–201 bp). The putative HlDnmt has Bromo Adjacent Homology (BAH) domains (interval 370–498 bp), Dnmt1-RFD (cytosine-specific DNA methyltransferase replication foci domain) at an interval of 19–148 bp, and Zinc finger (zf-CXXC) domain (interval 253–299 bp) which is present in a typical structure of Dnmt (Figure 2). The open reading frames of DsDnmt1 and HlDnmt are probably incomplete and it is speculated that they lack other domains at the N- and C-terminal, respectively.

#### Protein Secondary Structure Prediction

The prediction of the secondary structure of the DNA methyltransferase proteins was carried out and the results showed that the four DNA methyltransferases have mixed secondary structures (Supplementary Table 1). α-helix and random coiled loop account for a larger proportion of the structure, whereas β-sheet strand accounts for a relatively small proportion, which provides an important structural basis for protein functional implementation and conformation. Furthermore, the DNA binding sites and protein binding sites of the DNA methyltransferase proteins were predicted (Supplementary Table 2).

#### Protein Tertiary Structure (3D Structure) Prediction

**Figure 3:**
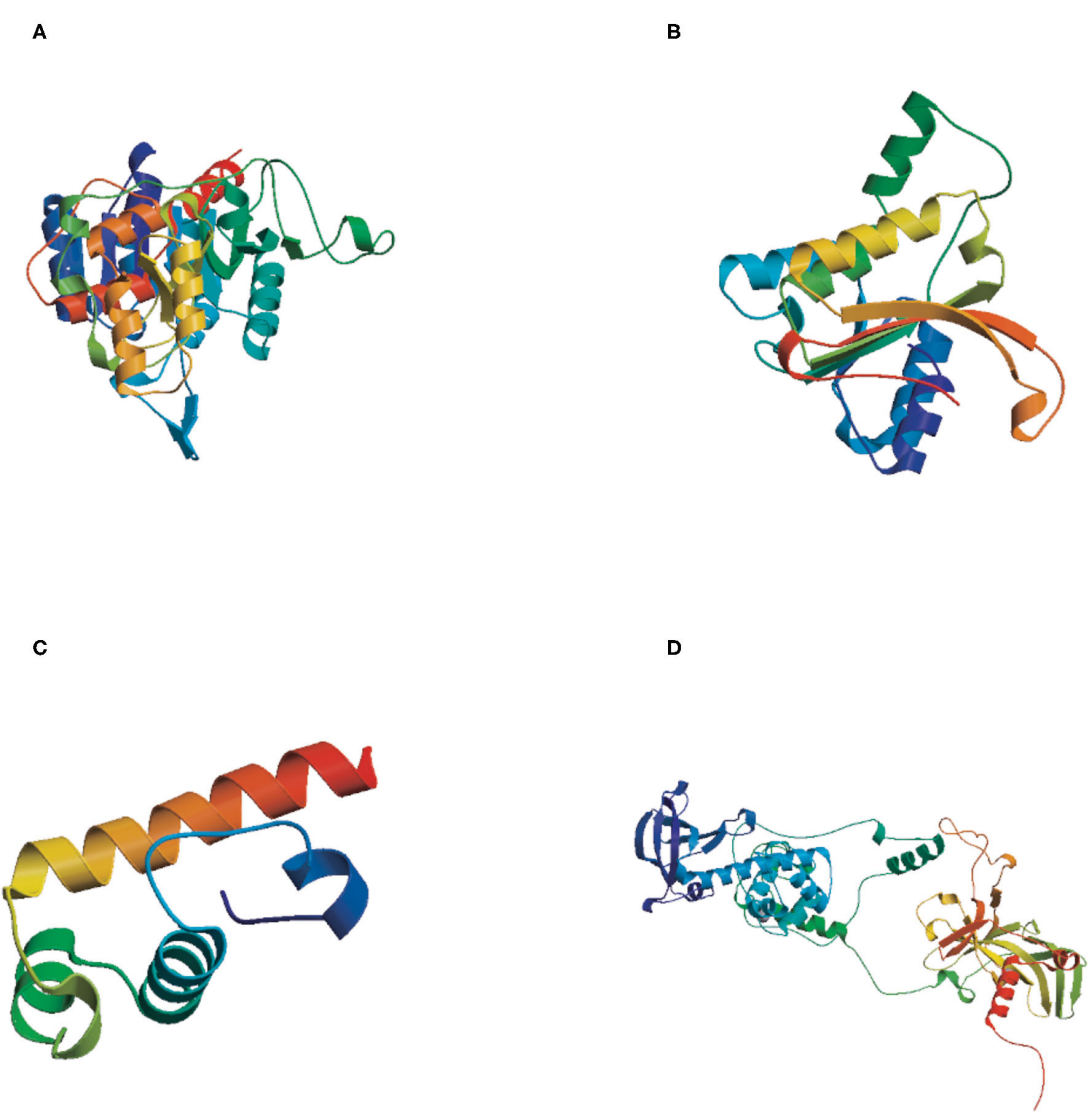
The tertiary structure (3D) of putative DNA methyltransferase proteins predicted by Swiss-Model. **(A)** DsDnmt, GMQE value = 0.65; **(B)** DsDnmt1, GMQE value = 0.79; **(C)** HlDnmt1, GMQE value = 0.10; **(D)** HlDnmt, GMQE value = 0.74.

The tertiary structures of the four DNA methyltransferase proteins were predicted by Swiss-Model (http://swissmodel.expasy.org/). The reliability range of the global model quality estimation (GMQE) is from 0 to 1. The GMQE values of DsDnmt, DsDnmt1, HlDnmt1, and HlDnmt proteins were 0.65, 0.79, 0.10, and 0.74, respectively. The tertiary structure prediction models of DsDnmt, DsDnmt1, and HlDnmt have higher confidence, except for HlDnmt1, possibly due to the lower homology of their amino acid sequences in the software database. The predicted DsDnmt contains one Ca^2+^ and one SAH (S-adenosyl-homocysteine) binding site, where SAH is the binding site of the methyl donor molecule SAM (S-adenosyl-L-methionine molecule); DsDnmt1 contains two Zn^2+^ binding sites. HlDnmt1 contains one Ca^2+^ binding site, whereas HlDnmt contains five Zn^2+^ and one SAH binding site (Figure 3).

#### Relative Expression of DNA Methyltransferase Genes Under Cold Treatments

**Figure 4:**
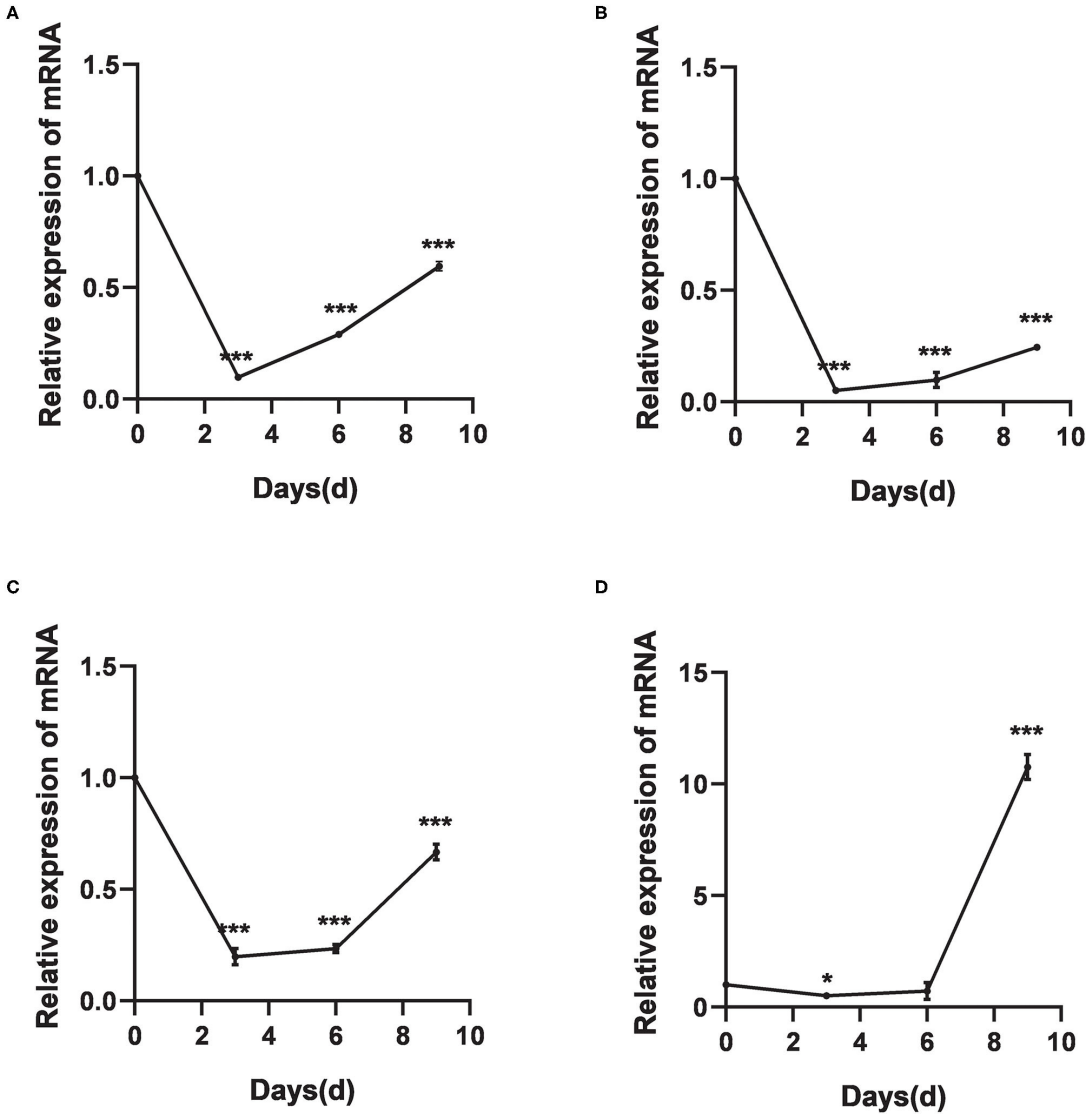
The relative expression of DNA methyltransferase mRNAs after cold treatments *vis-à-vis* the untreated group. **(A–D)** Relative expression of *DsDnmt* **(A)**, *DsDnmt1* **(B)**, *HlDnmt1* **(C)**, *HlDnmt* **(D)** measured using qRT-PCR. The results are expressed as the means (*n* = 3) ± SEM. The asterisks above bars indicate significant difference based on one-way ANOVA followed by Turkey's test, *P < 0.05, ***P < 0.01.

The relative expression levels of *HlDnmt1, DsDnmt1*, and *DsDnmt* decreased significantly after 3 days of cold treatment (*P* < 0.01), but increased with the extension of cold treatment time/days (Figures 4A–C). *HlDnmt* expression decreased significantly after 3 days of cold treatment (*P* < 0.05), but had a sharp significant increase (*P* < 0.01) after 9 days of cold treatment (Figure 4D).

#### The Efficiency of RNA Interference of DNA Methyltransferase

**Figure 5:**
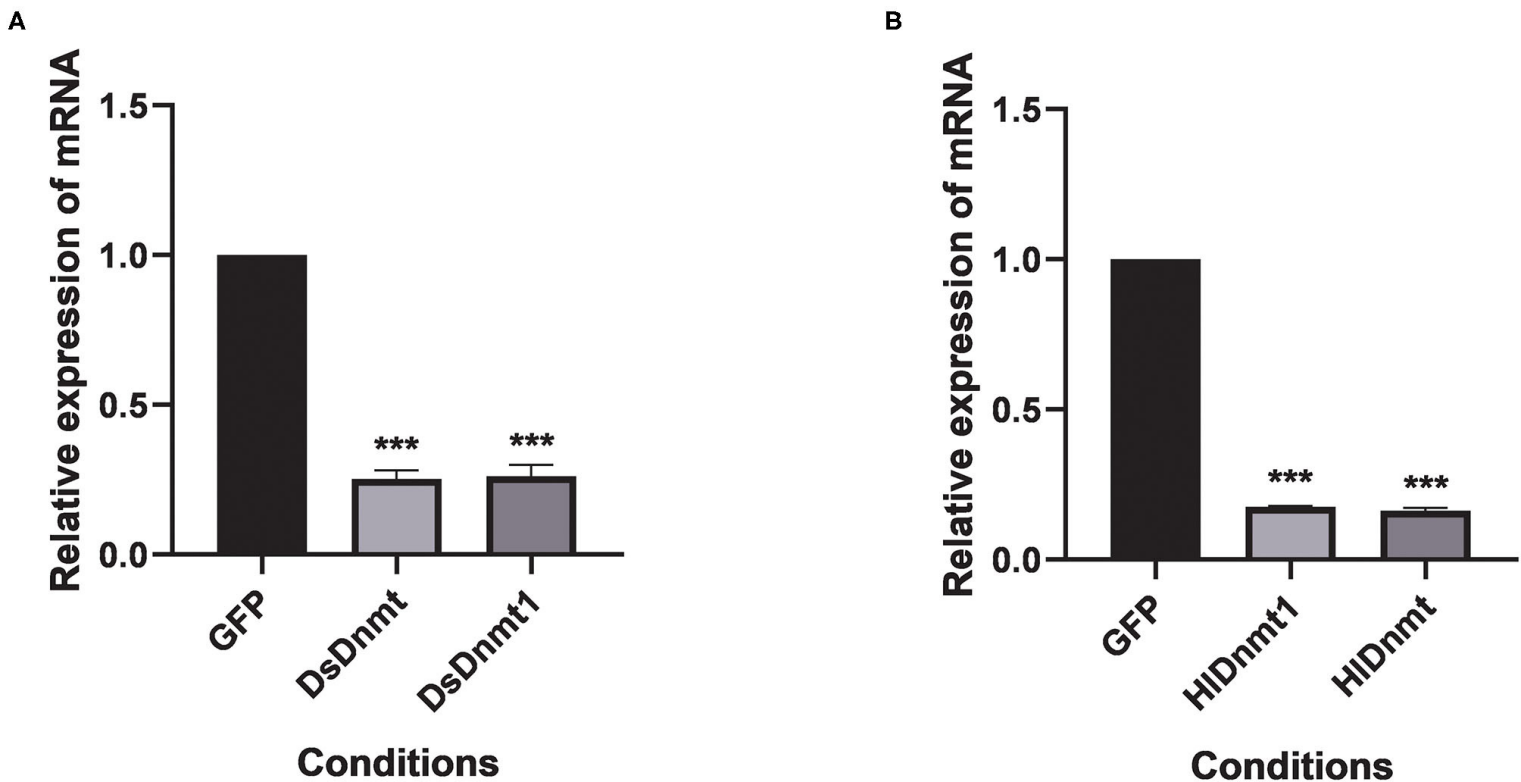
Effect of double-stranded RNA treatment on expression of *Dnmts* in *D. silvarum* **(A)** and *H. longicornis* **(B)**. The expressions of *Dnmts* were significantly decreased post 24 h after the injection of respective dsRNAs compared with expression in the controls. The results are expressed as the means (*n* = 3) ± SEM. The asterisks above bars indicate significant difference based on one way ANOVA followed by Turkey's test, ***P < 0.01. GFP represents the green fluorescent protein gene.

The relative expressions of the DNA methyltransferase gene were determined after RNAi. Results showed that the expression of the DNA methyltransferase genes was decreased significantly (*P* < 0.01) (Figure 5). Analysis of gene silencing efficiency showed that the average silencing efficiency of *DsDnmt* and *DsDnmt1* genes were 74.79 and 73.78%, respectively, whereas that of *HlDnmt1* and *HlDnmt* genes were 82.42 and 83.72%, respectively, indicating that the dsRNA injection of DNA methyltransferase gene had a significant silencing effect on the expression of the target gene.

#### Cold Tolerance of *D. silvarum* and *H. longicornis* After RNAi

**Figure 6:**
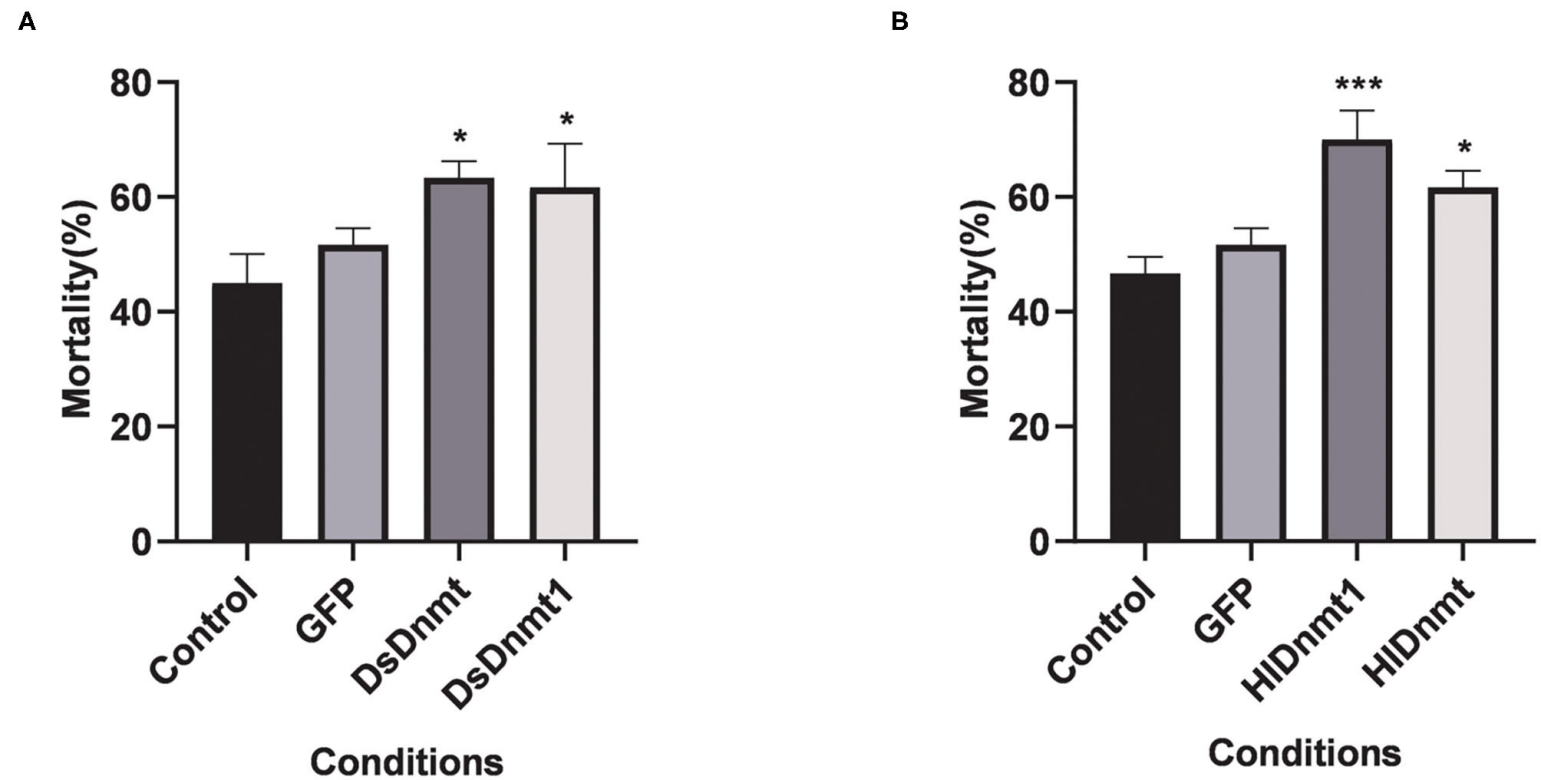
The effects on the cold tolerance of ticks after the knockdown of *Dnmts*. **(A)** The mortality rate of *D. silvarum* after cold treatment post *DsDnmt* and *DsDnmt1* knockdown. The mortality rate increased significantly in the ticks with Dnmts' knockdown in relation to the controls. **(B)** The mortality rate of *H. longicornis* after cold treatment post *HlDnmt1* and *HlDnmt* knockdown *H. longicornis*. The mortality rate increased significantly in the ticks with Dnmts' knockdown in relation to the controls. The results are expressed as the means (*n* = 3) ± SEM. The asterisks above bars indicate significant difference based on one-way ANOVA followed by Turkey's test, *P < 0.05, ***P < 0.01.

After the interference of DNA methyltransferases, the changes in cold tolerance of *D. silvarum* and *H. longicornis* were analyzed, and the role of DNA methyltransferase in the cold response of ticks was determined. The mortality rates of both tick species increased significantly (*P* < 0.05) (Figure 6). The resultant mortality rate from the knockdown of *DsDnmt, DsDnmt1*, and *HlDnmt* was more than 60%, while the resultant mortality from the knockdown of *HlDnmt1* was over 70%, indicating the important function of DNA methyltransferase in the cold tolerance of *D. silvarum* and *H. longicornis*.

#### Relative Expression of DNA Methyltransferase Proteins Under Different Cold Treatments

**Figure 7:**
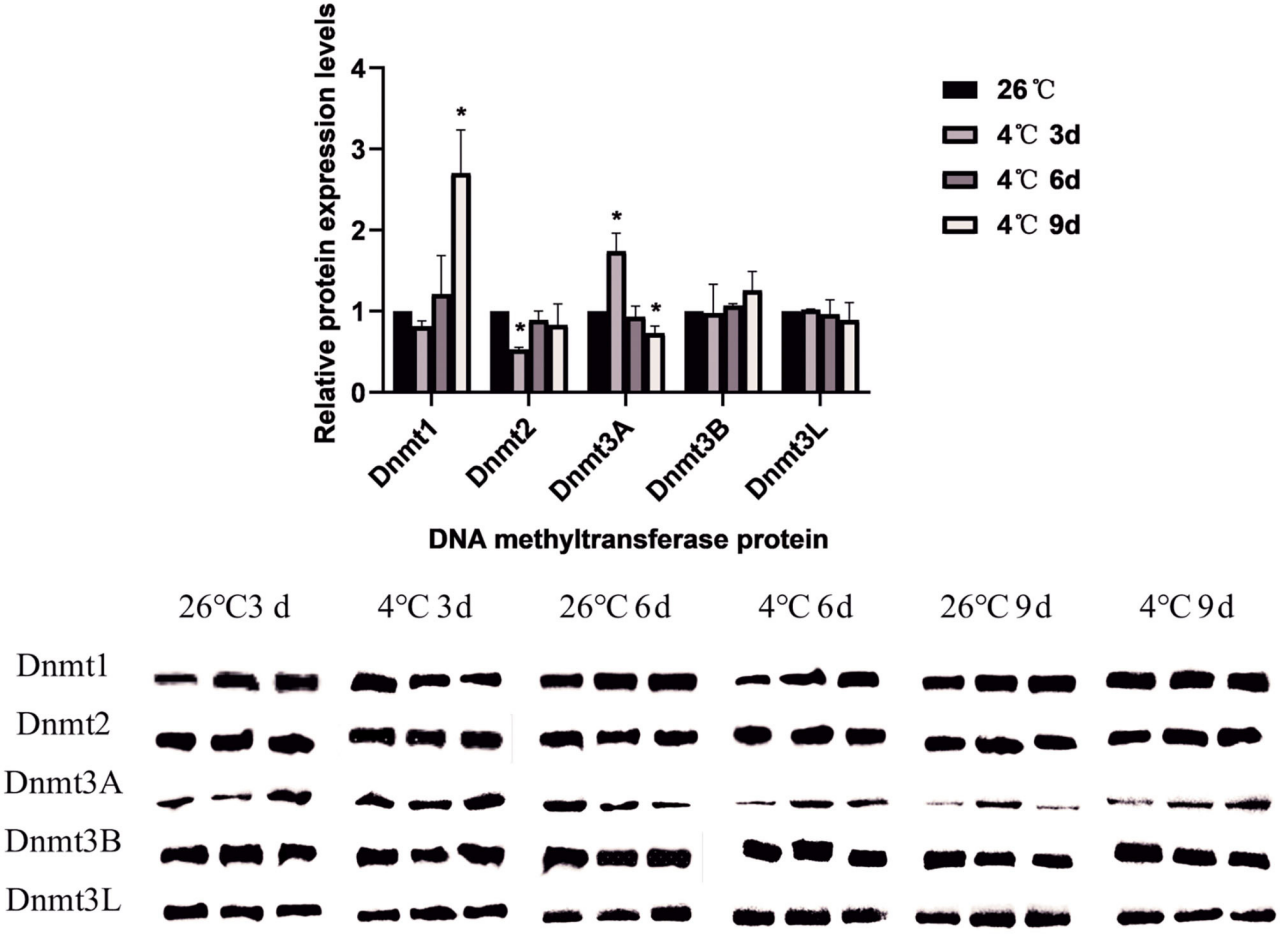
The relative protein expression of Dnmts in *D. silvarum* after cold treatment. The relative level of the expression of DNA methyltransferase proteins were calculated, and the expression in the control group served as the reference, which was set at 1. The results are expressed as the means (*n* = 3) ± SEM. The asterisks above bars indicate a significant difference based on one-way ANOVA followed by Turkey's test, *P < 0.05.

**Figure 8:**
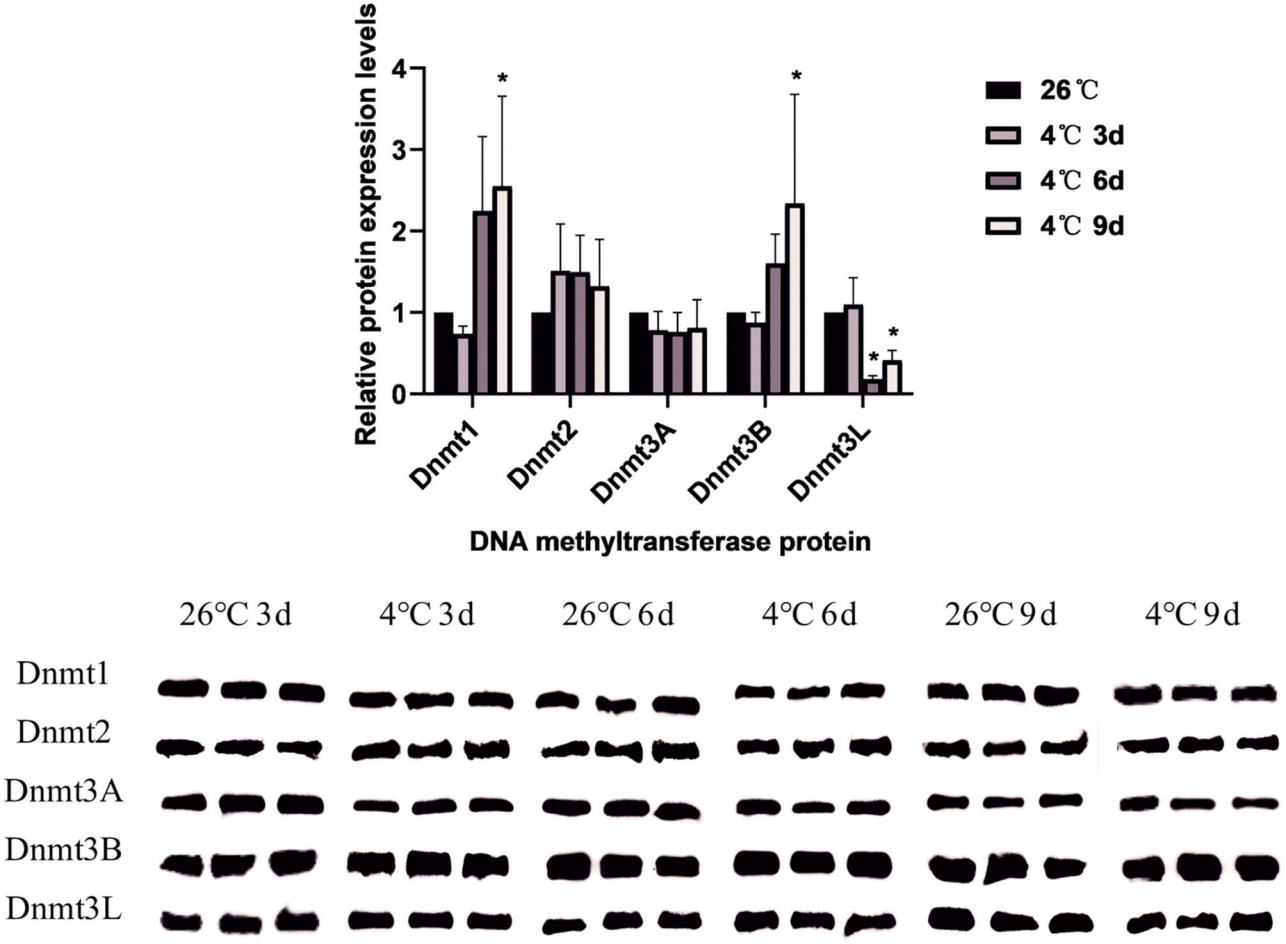
The relative protein expression of Dnmts in *H. longicornis* after cold treatment. The relative level of the expression of DNA methyltransferase proteins were calculated, and the expression in the control group served as the reference, which was set at 1. The results are expressed as the means (*n* = 3) ± SEM. The asterisks above bars indicate a significant difference based on one-way ANOVA followed by Turkey's test, *P < 0.05.

A total of 5 DNA methyltransferase-related proteins were selected (Dnmt1, Dnmt2, Dnmt3A, Dnmt3B, and Dnmt3L) as the target proteins which were determined depending on the commercially available specific antibody products. The results showed that the relative expression levels of various DNA methyltransferase proteins in the ticks increased or decreased to varying degrees after cold treatment. In *D. silvarum*, the protein expression of Dnmt1 and Dnmt2 changed significantly (*P* < 0.05). After cold treatment for 3 days, the protein expression was down-regulated, but with the extension of treatment, the expression was up-regulated. After cold treatment, the expression of Dnmt3A increased significantly (*P* < 0.05), and then gradually decreased. Dnmt3L showed a similar trend of change as Dnmt3A, but not to a significant level (Figure 7). In *H. longicornis*, the expression of Dnmt1 decreased after cold treatment for 3 days, and then gradually increased significantly (*P* < 0.05) after cold treatment for 9 days. Dnmt3B showed a similar trend as Dnmt1. The protein expression of Dnmt3L up-regulated initially after cold treatment before down-regulating with time, just as in *D. silvarum* ticks (Figure 8).

## Discussion

In the present study, four DNA methyltransferase genes were characterized from *D. silvarum* and *H. longicorni*s, and phylogenetic analysis revealed that they were clustered with the arthropod species into their corresponding taxonomic classes. The putative DsDnmt and DsDnmt1 share the same node with the sequences of the closely related species (*I. ricinus* and *I. scapularis*). Similarly, the putative HlDnmt1 and HlDnmt share the same node with the sequences of closely related *I. scapularis*. Ticks belong to a relatively independent branch and are closely related to other arachnids than the insects. Vertebrates such as mammals, birds, and fishes were placed in an independent branch indicating that their DNA methyltransferases are less closely related to those of ticks than mollusks and other marine organisms. Overall, these results confirm that DNA methyltransferases of ticks are good candidates for phylogenetic analysis at higher levels. However, a previous study could not find a common ancestry between putative *I. ricinus* DNA methyltransferase (IrDAMT) and other arachnid species, but it was rather placed in a sister branch of the cluster of Maxillopoda, Branchiopoda, and insect (38). Additionally, HlDnmt results suggest that they could be less suitable for a detailed study of the evolutionary relationship among arachnids/arthropods as none of its orthologs were found (apart from *I. scapularis*), presumably because of their non-existence in a publicly available database.

All the four putative proteins were predicted to have a nuclear localization, which is consistent with their hypothetical assignment to the methyltransferase group of enzymes. This was corroborated by the three putative *I. ricinus* methyltransferases—IrDNMT1, IrDNMT3, and IrDAMT (38). The instability index is a measure used to determine whether proteins will be stable in a test tube (53), and the index values above 40 are an indicator for unstable proteins, which is true for the four DNA methyltransferases in the present study. The conserved domain search revealed that putative DsDnmt and DsDnmt1 contain DNA cytosine methylase (Dcm) domain with multiple substrates- and DNA-binding sites. At the Dcm domain target sites (CCAGG and CCTGG), a methyl group is added to the 5th position of the cytosine base in the context of CpG dinucleotide by C-5 cytosine-specific DNA methylases (C5 Mtase); this plays a vital role in gene expression by either inhibiting the binding of transcription factors or the synthesis of methyl binding protein and related chromatin remodeling factors (54, 55). HlDnmt1 contains the DMAP1 (DNA methyltransferase 1-associated protein) domain and SANT domain. DMAP1 possesses intrinsic transcriptional inhibitory activity and could play an important role in the development and maintenance of the integrity of methylation via interaction with Dnmt1 (56), whereas the SANT domain contains a putative DNA-binding site (57). Moreover, some interesting findings showed that the SANT domain was involved in the activities of ATP-dependent chromatin remodeling enzymes, as well as in the histone acetyltransferase and deacetylase complexes (57–59). The putative HlDnmt has BAH domain, Dnmt1-RFD at the interval of 19–148 bp, and Zinc finger-CXXC (zf-CXXC) domain. BAH domain is responsible for linking DNA methylation, replication, and transcriptional regulation (37, 60). The functions of the BAH domain in chromatin biology are versatile including taking part in the protein-protein interaction that mediates gene silencing, recognition of methylated histones, and direct interaction with nucleosome (61). Dnmt1-RFD is located at the longer N-terminal region of Dnmt1 which targets the replication foci (62). Specifically, it ensures that the Dnmt1 protein is targeted toward the replication foci in a non-catalytic fashion, which allows it to methylate the correct residues, and has been associated with the conversion of critical histone lysine moieties (63). Of note, Dnmt1-RFD binds to HDACs (histone deacetylases) and DMAP1 in a manner that can mediate transcriptional repression (63). The zf-CXXC domain contains eight conserved cysteine residues (present in the typical structure of Dnmts) which bind to Zn^2+^ ions and non-methylated CpG dinucleotides (64). Although it was generally acknowledged that methylated CpGs (cytosine guanine dinucleotides) are specifically recognized by proteins that encode MBDs (methyl-CpG-binding domains) (65, 66), it has also been proposed that non-methylated CpG dinucleotides could present a protein-binding site (64). This was made possible by the identification of non-methylated CGBP (CpG-binding protein) whose DNA-binding capacity is dependent on a cysteine-rich zf-CXXC domain (67).

Temperature is one of the most important factors that greatly influence the development, growth, geographical distribution, abundance, and physiology of organisms (68, 69). Organisms respond to changes in environmental temperature by activating stress response mechanisms. Studies suggest that epigenetic enzymes play a vital role in arthropod cold hardiness (70). To the best of our knowledge, this is the first report of the response of DNA methyltransferases of *D. silvarum* and *H. longicornis* ticks to cold stress. Several studies with other species have demonstrated the relationship between DNA methylation, a vital environment-induced epigenetic phenomenon, and variations in temperature (40, 71–73). For example, there was a significant change in the expression level of *Dnmt3* in the *Senegalese sole* (fish) after short-term exposure to embryonic temperatures (42). In the present study, the first 3 days of cold exposure were marked with a significant decrease in the expression levels of all the Dnmt genes but increased significantly with the prolongation of days of cold treatment. A similar trend was observed in the armyworm (*Mythimna separata*) where a decreased pattern of expression was observed after short exposures to various temperatures (73). A sharp significant increase in the expression levels of HlDnmt was observed after 9 days of cold exposure (4°C). This was corroborated by the significant up-regulation of the expression levels of *Bemisia tabaci* DNA methyltransferase genes (*BtDnmt3*) after exposure to low temperatures ranging from 0 to 20°C (74). A possible explanation could be that for the first few days of cold exposure, the ticks employ other physiological strategies such as cryoprotective dehydration (28), the modification of body fluid constituents (27), and the utilization of numerous potentially protective molecules in their body (26) before the full activation of the activities of the Dnmts as the cold stress prolongs. Additionally, the same pattern of expression was observed via western blot where the relative expression of various DNA methyltransferase proteins in the ticks decreased or increased to varying degrees after cold treatment. The protein expression level was down-regulated after 3 days of cold treatment but was up-regulated with the extension of treatment time for both the Dnmts of *D. silvarum* and *H. longicornis*. Similarly, a study observed that cold and subzero temperature exposure in *Eurosta solidaginis* (goldenrod gall fly) resulted in the upregulation of several DNA methyltransferases (70). The down-regulation and up-regulation due to temperature variations is a confirmation of the thermal plasticity of *Dnmt* expression which is reflected in the corresponding protein expression. Ticks are remarkably plastic and can inhabit diverse ecological niches, from the tropics to the polar regions of the planet (2, 75). Like insects, ticks are ectotherms that are sensitive to external temperature variations (76). The cold hardiness of ticks that enables them to overwinter could be a function of epigenetic modifications such as DNA methylation that might occur rapidly in response to large-scale temperature changes (40).

Due to the vital role of DNA methyltransferases in different biological processes, the knockdown of *DsDnmt, DsDnmt1, HlDnmt1*, and *HlDnmt* was carried out to identify their functions by assessing the mortality rate after the exposure of *D. silvarum* and *H. longicornis* ticks to sublethal low temperatures of −22 and −14°C, respectively, for 2 h. Interestingly, the mortality rates of the two tick species significantly increased which underscores the impact of DNA methyltransferases on the cold tolerance of ticks. This result was corroborated by the resultant significant mortality of armyworms (whose *MsDnmt1* genes have been knocked down) after exposure to a low temperature (73). Previous studies have demonstrated the effect of extreme temperatures on arthropod development which include reduced fecundity, deformity, and mortality (77–79). It is noteworthy that ticks, which are widely distributed globally, can adapt to different climatic regions and they might possess unique adaptability mechanisms. One of these adaptability mechanisms could be mediated by DNA methyltransferases. DNA methylation, a vital epigenetic mechanism induced by the environment, can serve as a potential link between phenotypic variability and temperature variation, which is reprogrammed by Dnmts (40).

## Conclusion

This study characterized DNA methyltransferases in *D. silvarum* and *H. longicornis*, and the results suggest that they contribute to the cold tolerance of ticks. The above findings highlighted the functional implications of DNA methyltransferases during cold stress of ticks and thus could advance our understanding of the epigenetic regulation of cold acclimation and adaptation of ticks.

## Data Availability Statement

The datasets presented in this study can be found in online repositories. The names of the repository/repositories and accession number(s) can be found in the article/Supplementary Material.

## Author Contributions

DA wrote the manuscript. MiZ, XS, SZ, MeZ, TW, and DA conducted the literature, performed the experiments, and prepared all the figures and tables. ZY, AM, and JL reviewed and edited the manuscript. All authors contributed to the article and approved the submitted version.

## Conflict of Interest

The authors declare that the research was conducted in the absence of any commercial or financial relationships that could be construed as a potential conflict of interest.

## Publisher's Note

All claims expressed in this article are solely those of the authors and do not necessarily represent those of their affiliated organizations, or those of the publisher, the editors and the reviewers. Any product that may be evaluated in this article, or claim that may be made by its manufacturer, is not guaranteed or endorsed by the publisher.

## Supplementary Material

Supplementary Figure 1 Agarose gel illustrating the results of PCR amplification of DNA methyltransferase genes. **(A)** Gene 1 (*DsDnmt*) fragments of *D. silvarum*; **(B)** Gene 2 (*DsDnmt1*) fragment of *D. silvarum*; **(C)** Gene 1 (*HlDnmt1*) fragments of *H. longicornis*; **(D)** Gene 2 (*HlDnmt*) fragments of *H. longicornis*.

Supplementary Figure 2 Amino acid sequence homologous alignment results of DNA methyltransferases. **(A)** DsDnmt; **(B)** DsDnmt1; **(C)** HlDnmt1; **(D)** HlDnmt.

Supplementary Figure 3 Hydrophobicity prediction of the amino acid sequence of DNA methyltransferases. **(A)** DsDnmt; **(B)** DsDnmt1; **(C)** HlDnmt1; **(D)** HlDnmt.

Supplementary Table 1 The secondary structure prediction of the DNA methyltransferase proteins of *Dermacentor silvarum* and *Haemaphysalis longicornis*.

Supplementary Table 2 The binding sites prediction of the DNA methyltransferase protein.

## Abbreviations

Leu: Leucine
Ser: serine
Pro: proline
Arg: arginine
Asp: aspartic acid
Glu: glutamic acid
Lys: lysine
Gly: glycine
Ala: alanine
Trp: tryptophan
Cys: cysteine
Val: valine
Gln: glutamine
Phe: phenylalanine
His: histidine.
