# Supplementary figures and images for: DNA Methyltransferases Contribute to Cold Tolerance in Ticks Dermacentor silvarum and Haemaphysalis longicornis (Acari: Ixodidae)
Source: Front Vet Sci. 2021 Aug 26;8:726731. doi: 10.3389/fvets.2021.726731 (PMC8426640; doi:10.3389/fvets.2021.726731)

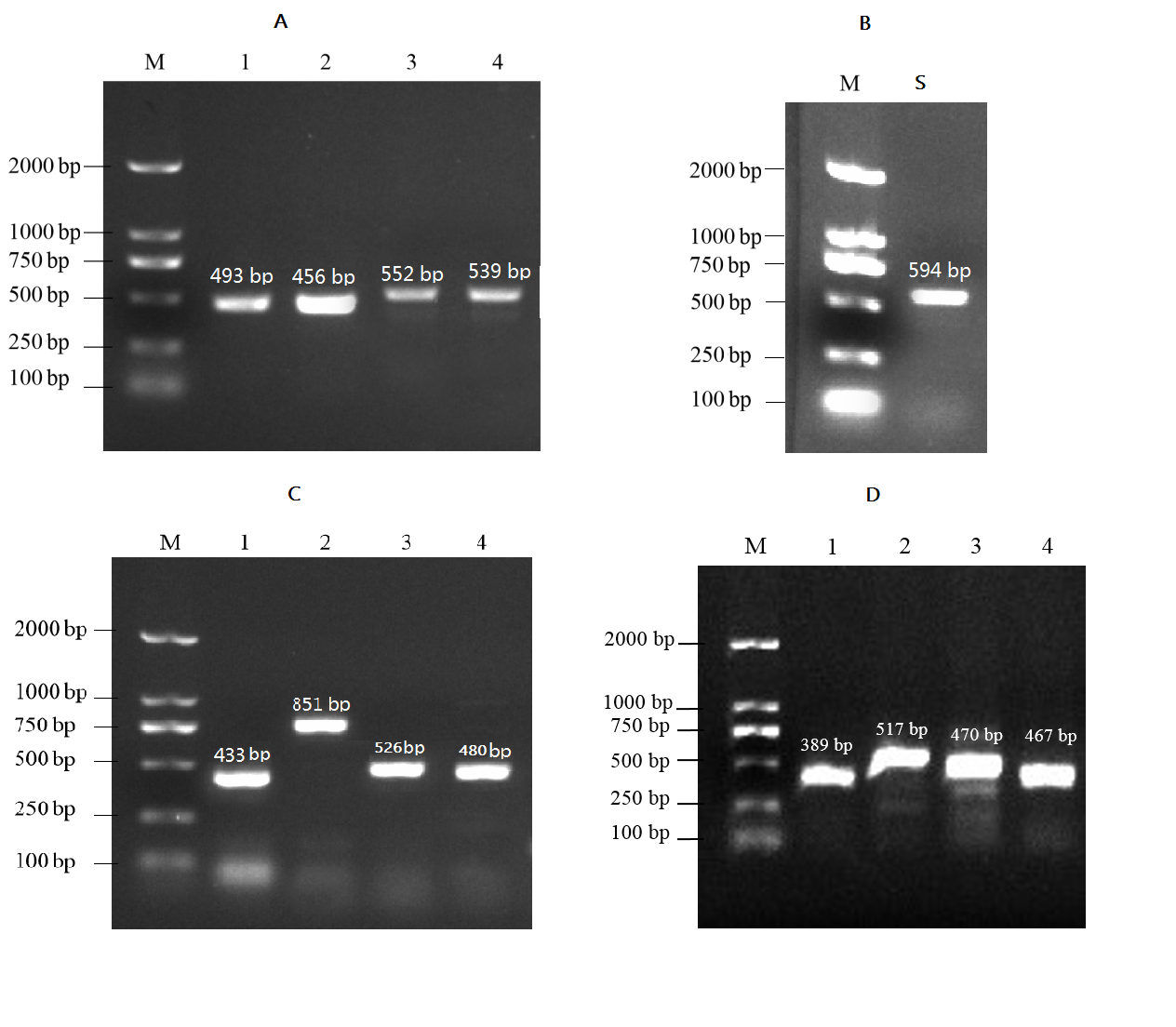

Supplement: Supplementary Figure 1 — Agarose gel illustrating the results of PCR amplification of DNA methyltransferase genes. (A) Gene 1 (DsDnmt) fragments of D. silvarum; (B) Gene 2 (DsDnmt1) fragment of D. silvarum; (C) Gene 1 (HlDnmt1) fragments of H. longicornis; (D) Gene 2 (HlDnmt) fragments of H. longicornis. [file Image_1.TIF]

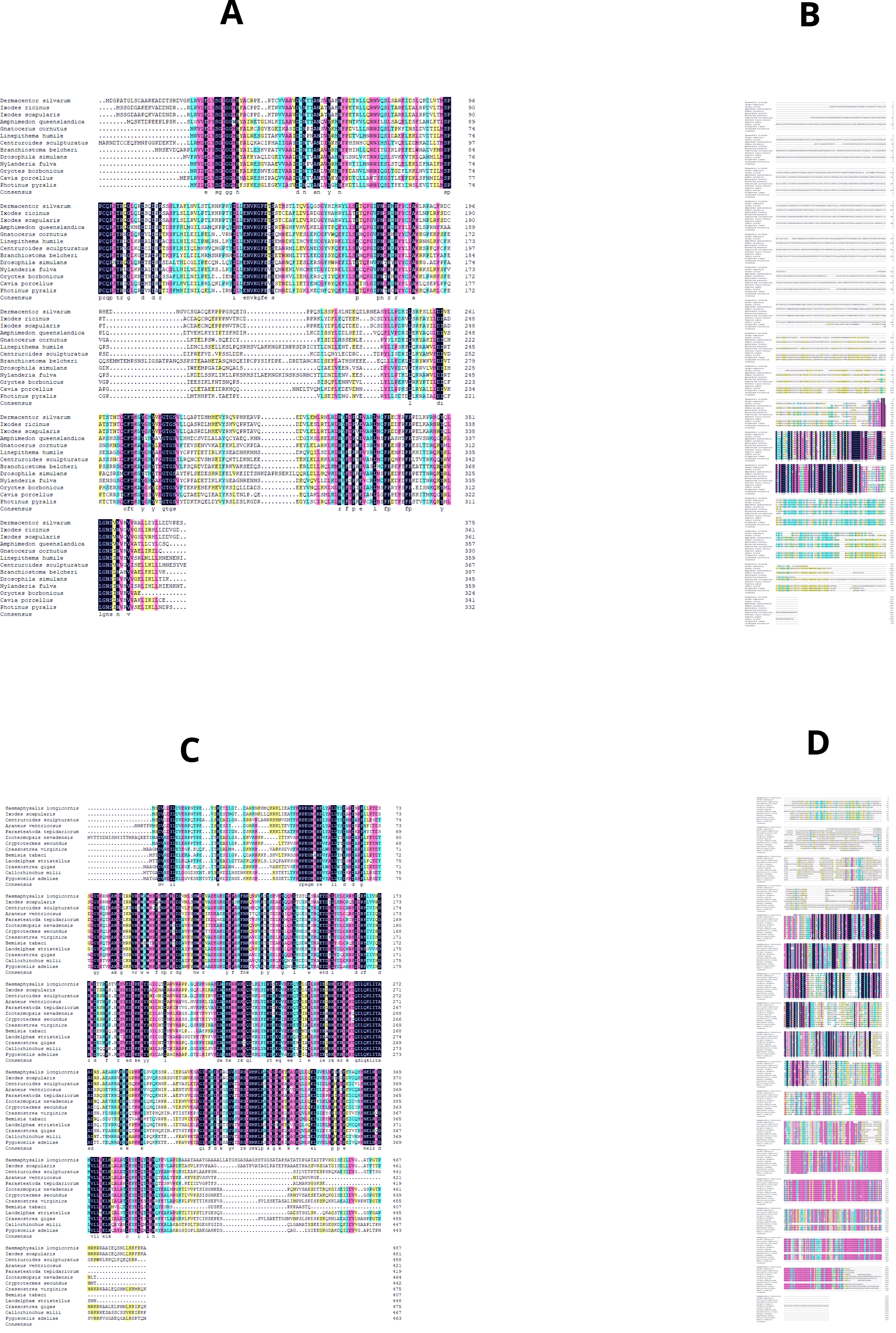

Supplement: Supplementary Figure 2 — Amino acid sequence homologous alignment results of DNA methyltransferases. (A) DsDnmt; (B) DsDnmt1; (C) HlDnmt1; (D) HlDnmt. [file Image_2.TIF]

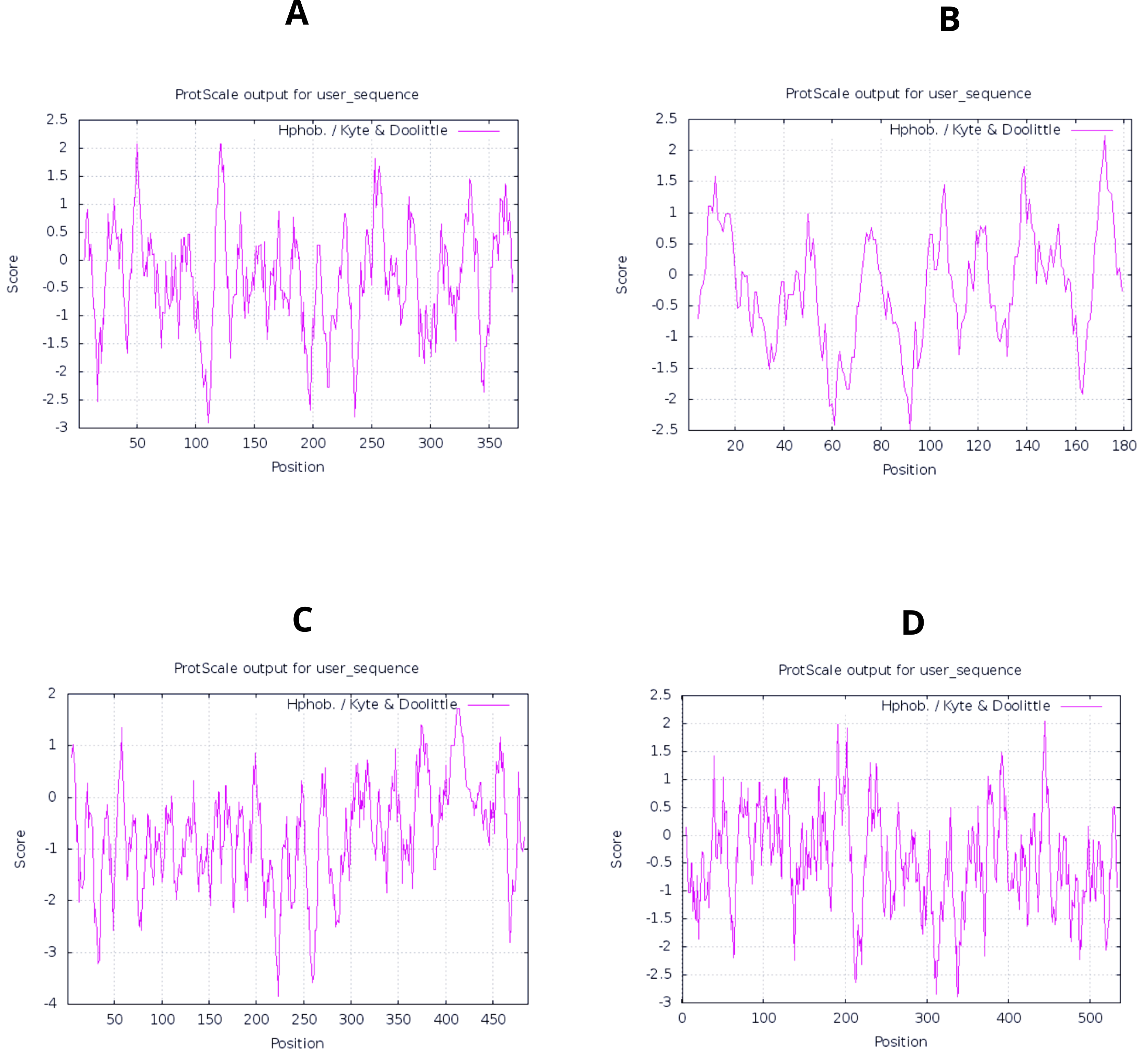

Supplement: Supplementary Figure 3 — Hydrophobicity prediction of the amino acid sequence of DNA methyltransferases. (A) DsDnmt; (B) DsDnmt1; (C) HlDnmt1; (D) HlDnmt. [file Image_3.TIF]
