# Supplementary material for: DNA Methyltransferases Contribute to Cold Tolerance in Ticks Dermacentor silvarum and Haemaphysalis longicornis (Acari: Ixodidae)
Source: Front Vet Sci. 2021 Aug 26;8:726731. doi: 10.3389/fvets.2021.726731 (PMC8426640; doi:10.3389/fvets.2021.726731)
Supplement: Supplementary Table 1 — The secondary structure prediction of the DNA methyltransferase proteins of Dermacentor silvarum and Haemaphysalis longicornis. [file Table_1.docx]

| **Supplementary Table S1:** The secondary structure prediction of the DNA methyltransferase proteins of *Dermacentor silvarum* and *Haemaphysalis longicornis* | | | | | | |
| --- | --- | --- | --- | --- | --- | --- |
| **Protein name** | **Composition (%)** | | | **Solvent accessibility (%)** | | |
|  | α-Helix | β-sheet | Random coil | Exposed | Buried | Intermediate |
| DsDnmt | 21.87 | 8.53 | 69.60 | 44.00 | 48.27 | 7.73 |
| DsDnmt1 | 24.04 | 15.85 | 60.11 | 51.37 | 38.25 | 10.38 |
| HlDnmt1 | 30.80 | 3.49 | 65.71 | 67.56 | 25.05 | 7.39 |
| HlDnmt | 9.50 | 15.08 | 75.42 | 45.44 | 46.18 | 8.38 |
