# Supplementary material for: DNA Methyltransferases Contribute to Cold Tolerance in Ticks Dermacentor silvarum and Haemaphysalis longicornis (Acari: Ixodidae)
Source: Front Vet Sci. 2021 Aug 26;8:726731. doi: 10.3389/fvets.2021.726731 (PMC8426640; doi:10.3389/fvets.2021.726731)
Supplement: Supplementary Table 2 — The binding sites prediction of the DNA methyltransferase protein. [file Table_2.docx]

| **Supplemetary Table S2:** The binding sites prediction of the DNA methyltransferase protein | | | | |
| --- | --- | --- | --- | --- |
| **Binding site** | **Protein name** | | | |
|  | DsDnmt | DsDnmt1 | HlDnmt1 | HlDnmt |
| DNA binding site | 114, 346 | 2-4 | 469 | - |
| RNA binding site | 145, 146 | 2, 4 | 469-471 | - |
| Protein binding site | 1, 103, 198 | 30, 56, 58 | 1, 28-31 | 369 |
|  | 200, 217 | 59, 64, 65 | 62, 86-88 |  |
|  | 218, 239 | 67-69, 88-99 | 118, 178-181, |  |
|  | 252, 271-273, | 129, 131, 134, | 230, 255, 260 |  |
|  | 276-279, 298, | 143, 145, 178- | 284, 477 |  |
|  | 300, 302-305, | 180 |  |  |
|  | 309, 318 |  |  |  |
